# Sleep disturbances in an arctic population: The Tromsø Study

**DOI:** 10.1186/1472-6963-8-117

**Published:** 2008-05-29

**Authors:** Arne Fetveit, Jørund Straand, Bjørn Bjorvatn

**Affiliations:** 1 Department of General Practice and Community Medicine, University of Oslo, PO Box 1130 Blindern, 0317 Oslo, Norway; 2 Department of Public Health and Primary Health Care, University of Bergen, Bergen, Norway; 3 Norwegian Competence Center for Sleep Disorders, Haukeland University Hospital, Bergen, Norway

## Abstract

**Background:**

Prevalence estimates for insomnia range from 10 to 50% in the adult general population. Sleep disturbances cause great impairment in quality of life, which might even rival or exceed the impairment in other chronic medical disorders. The economic implications and use of health-care services related to chronic insomnia represent a clinical concern as well as a pronounced public health problem. Hypnotics are frequently prescribed for insomnia, but alcohol and over-the-counter sleep aids seem to be more widely used by insomniacs than prescription medications. Despite the complex relationship between insomnia and physical and mental health factors, the condition appears to be underrecognized and undertreated by health care providers, probably due to the generally limited knowledge of the causes and natural development of insomnia.

**Methods/Design:**

The Tromsø Study is an ongoing population-based cohort study with five previous health studies undertaken between 1974 and 2001. This protocol outlines a planned study within the sixth Tromsø Study (Tromsø VI), aiming at; 1) describing sleep patterns in a community-based sample representative of the general population of northern Norway, and 2) examining outcome variables of sleep disturbances against possible explanatory and confounding variables, both within a cross-sectional approach, as well as retrospectively in a longitudinal study – exploring sleep patterns in subjects who have attended two or more of the previous Tromsø studies between 1974 and 2009. First, we plan to perform a simple screening in order to identify those participants with probable sleep disturbances, and secondly to investigate these sleep disturbances further, using an extensive sleep-questionnaire. We will also collect biological explanatory variables, i.e. blood samples, weight, height and blood pressure. We plan to merge data on an individual level from the Tromsø VI Study with data from the Norwegian Prescription Database (NorPD), which is a national registry including data for all prescription drugs issued at Norwegian pharmacies. Participants with sleep disturbances will be compared with pair-matched controls without sleep disturbances.

**Discussion:**

Despite ongoing research, many challenges remain in the characterization of sleep disturbances and its correlates. Future mapping of the biological dimensions, natural history, as well as the behavioral and drug-related aspects of sleep disturbances in a representative population samples is clearly needed.

## Background

Insomnia is the subjective complaint of insufficient sleep quantity or quality leading to impaired daytime functioning, usually linked to difficulties in falling asleep, trouble staying asleep, waking up too early in the morning or non-restorative sleep [1]. The real prevalence of insomnia is not known, but prevalence estimates, which in part are influenced by diagnostic definitions, range from 10 to 50% in the adult population [2].

Insomnia is classified as transient (no more than a few nights), acute (less than 3 to 4 weeks), and chronic (more than 3 to 4 weeks). Transient or acute insomnia usually occurs in people with no history of sleep disturbances and is often related to an identifiable cause [3]. Chronic insomnia is further divided into primary and secondary conditions, depending on the cause [4]. When the complaint of insomnia is not caused by other medical, psychiatric or medication-related disorders, it is considered to be primary in nature [4]. It is estimated that primary insomnia accounts for approximately 25% of all chronic insomnia [5]. Recently, The American National Institutes of Health State-of-the-Science Conference suggested that insomnia should be conceptualized as a disorder in and of itself, which is or is not comorbid with other disorders – rather than drawing conclusions about insomnia's primary or secondary status [6].

Excessive daytime sleepiness is a key symptom of any type of disturbed sleep. Sleepiness is defined by recurrent episodes of drowsiness or involuntary dozing that arise mainly in sedentary situations. Sleepiness should not be confused with fatigue or tiredness. Although subjective reports of sleepiness are common in people with insomnia, many studies report normal or even heightened levels of daytime alertness among insomniacs, e.g. as measured with the Epworth sleepiness scale (ESS) [7,8] or the Multiple Sleep Latency Test (MSLT) [9-12]. On the other hand, insomnia patients usually experience more fatigue than patients with other sleep disturbances, such as obstructive sleep apnea, narcolepsy or periodic limb movements during sleep [7,13].

A wide range of factors are associated with chronic insomnia (Table 1). Precipitants of acute insomnia may include acute medical illness, hospitalization, changes in the sleeping environment, medications, jet lag, and acute or recurring psychosocial stressors [3]. Chronic insomnia is often linked to or associated with various underlying medical, behavioral, and environmental conditions [3,14,15], and various medications [14-16]. Especially mental disorders and discomfort are frequently reported by insomniacs. In a survey of 811 respondents, 40% of those with insomnia (compared with 16% of those without), met the criteria for one or more psychiatric diagnoses [17]. Although it is difficult to discern whether a psychiatric disorder precipitates insomnia or whether insomnia makes an individual vulnerable to the emergence of a psychiatric disorder, investigations indicate an increased risk for new onset of depression, anxiety disorders, and substance abuse in people with persistent insomnia [18]. Additionally, insomnia may be precipitated by factors like stress and perpetuated by behavioral factors, or unstable sleep schedules. Shift work or other lifestyle factors that disrupt circadian rhythm increase the risk of sleep disturbances [19].

**Table 1:** Factors related to chronic insomnia

Specific sleep disturbances:
• Circadian rhythm disorders:
◦ Advanced sleep-phase syndrome
◦ Delayed sleep-phase syndrome
• Sleep apnea (obstructive, central, or mixed)
• Restless leg syndrome
• Periodic limb movement disorders (nocturnal myoclonus)
• Parasomnias, i.e. REM-sleep-behavior-disorder
Physical illness:
• Pain: arthritis, musculoskeletal pain, other painful conditions
• Cardiovascular: heart failure, nocturnal breathlessness, nocturnal angina
• Pulmonary: chronic obstructive pulmonary disease, allergic rhinitis (nasal obstruction)
• Gastrointestinal: gastroesophageal reflux disease, peptic ulcer disease, constipation, diarrhea, pruritus ani
• Urinary: nocturia, incomplete bladder emptying, incontinence
• Central nervous system: stroke, Parkinson disease, Alzheimer disease, seizure disorder
• Psychiatric illness: anxiety, depression, psychosis, dementia, delirium
• Pruritus
• Menopause (hot flushes)
Behavioral: daytime nap, early retirement to bed, use of bed for other activities (eg, reading and watching television), heavy meals, lack of exercise, and sedentary lifestyle
Environmental: noise, light and other disturbances, extreme temperatures, uncomfortable bedding, and lack of exposure to sunlight
Medications:
• Central nervous system stimulants: sympathomimetics, caffeine, nicotine, amphetamines, ephedrine, phenytoin
• Antidepressants: bupropion, selective serotonin reuptake inhibitors, venlafaxine
• Anti-Parkinsonian agents: levodopa
• Bronchodilators: theophylline
• Cardiovascular: B-blockers, diuretics
• Histamines, H_2 _blockers: cimetidine
• Anticholinergics
• Corticosteroids
• Alcohol
• Herbal remedies
• Stimulant laxative

REM = rapid eye movement.

An association between elevated body mass index (BMI) and short sleep duration has been demonstrated in recent years [20]. A possible causality between short sleep duration and the development of diabetes mellitus (DM) has also been explored. Gottlieb et al. [21] reported that short sleep duration is associated with DM and impaired glucose tolerance (IGT) in community-dwelling middle-aged and older adults. This association persisted after adjustment for known DM risk factors. Sleep restriction to four hours of sleep per night increased blood pressure, decreased parasympathetic tone, increased evening cortisol and insulin levels, and promoted increased appetite, possibly through the elevation of ghrelin, a pro-appetitive hormone, and decrease in levels of leptin [22-24]. The correlation between short sleep duration with DM and IGT may partly explain the association between short sleep duration and coronary heart disease [25] and all over mortality [26-28].

Sleep patterns change throughout life. It seems as if ageing makes it more difficult to stay asleep at night and to stay awake during the day [29]. Total sleep time during the 24-hour period appears to decline with advancing age, accompanied by a redistribution of sleep – with decreased nocturnal sleep and increased daytime napping [29]. Progressive physical inactivity, dissatisfaction with social life, and presence of physical and mental problems may be the most predictive factors of insomnia in the elderly [30]. Opposed to healthy elderly, sleep duration during the 24-h day in demented nursing home patients seems to increase according to the degree of dementia [31]. Some researchers claim that ageing probably is the single most important factor that determines human sleep – more so than gender and even most physical and mental illnesses [32]. However, challenging findings of *no *reduction of nocturnal sleep with age in healthy elderly have also been presented, suggesting that insomnia in the elderly is not related to ageing itself [33-35].

It is known that hypnotics are frequently prescribed for insomnia [36]. However, alcohol and over-the-counter (OTC) medications seem to be more widely used by insomniacs than prescription medications [37], perhaps due to the fact that most insomniacs do not discuss their sleep problems with their doctor [38]. In a general population sample of 2.181 adults aged 18 to 45 years, Johnson et al. [37] found that 25.9% had used any substance as a sleep aid in the past 12 months. Approximately 18% reported using medications (57% of these being OTC-drugs), and 13% reported using alcohol to fall asleep. The choice of substance used as a sleep aid seems to be affected by sociodemographic characteristics: Most chronic benzodiazepine users tend to be elderly [39], and more men than women use alcohol to induce sleep. Rokstad et al. [40] examined the prescribing patterns among general practitioners (GPs) in a Norwegian county in relation to the patients' age, gender and the diagnosis for prescribing, and found that insomnia was among the most commonly recorded diagnosis for prescribing. Most drugs have not been sufficiently studied to determine their primary effects on sleep and waking behavior [41]. Even when the effects of a drug are known, the medication may act differently in normal individuals and individuals who are ill [41].

The economic implications and use of health-care services related to chronic insomnia represent a major problem. Costs related to chronic insomnia do not only include the direct costs of health-care, but also the indirect costs related to absence from work, diminished productivity, accidents, and other health problems that are, at least in part, secondary to insomnia [42-44].

Compared to other sleep disturbances, such as narcolepsy and sleep apnea, the understanding of the basic pathophysiology of insomnia has lagged behind [45]. This discrepancy probably stems from the heterogeneous nature of insomnia. On one hand, insomnia may be a primary condition with a pathophysiology, like a general state of hyperarousal – which includes changes like increased levels of catecholamines [46], increased basal metabolic rate [47], increased body temperature [48], altered heart rate [47], increased level of central nervous system (CNS) metabolic rate [49], and elevated electroencephalograph activity [50,51]. On the other hand, insomnia may be a co-existing condition with numerous physical and mental disorders.

Further knowledge of the impact of latitude, daytime illumination and season, on the prevalence of insomnia is needed [52]. The term "midwinter insomnia" has been applied for the seasonal type of insomnia observed in arctic areas during the dark periods of the year [53], but the possible consequences of annual variations in environmental light on sleep are still not settled [52,54], and make arctic sleep studies especially relevant.

Semantic as well as definition confusions are present in many epidemiological sleep studies. This situation probably rises from the fact that no single and clear definition exists of insomnia and how to assess it [33]. As a result, very different and non-comparable sleep assessment tools exist – making it is almost impossible to compare and summarize studies.

Our knowledge of insomnia is still limited, especially regarding its evolution and consequences in the general population [33]. Future sleep research is in need of validated measurements of sleep complaints, in order to provide common metrics for describing insomnia and insomniacs in epidemiological studies.

Below, we describe the protocol for a longitudinally, retrospective cohort survey and a cross-sectional study in a large representative adult community sample in northern Norway (The Tromsø Study). Using validated sleep assessment tools, our aim is to describe and analyze sleep disturbances, with their correlating and predisposing factors.

### Aims and objectives

The Tromsø Study is a large ongoing population-based cohort study with five previous health studies undertaken between 1974 and 2001 (Table 2). This protocol outlines a planned study in the sixth Tromsø Study (Tromsø VI), which will be carried out in 2008–2009.

**Table 2:** Participation in five previous Tromsø Studies, 1974 to 2001

			**No. invited**		**No. attending**		**Attendance (%)**	
			
**Study**	**Year**	**Year of birth**	**Men**	**Women**	**Men**	**Women**	**Men**	**Women**
Tromsø I	1974	1927–1956	8 867		6 595		83	
Tromsø II	1979–1980	1925–1959^1^	11 481	9 959	8 477	8 144	73.8	81.8
Tromsø III	1986–1987	1925–1966^1^	14 539	12 877	10 413	10 189	71.6	79.1
Tromsø IV	1994–1995	<1970	18 480	19 078	12 865	14 293	69.6	74.9
Tromsø V	2001	<1972^2^	4 636	5 717	3 511	4 619	75.7	80.8

^1^From 1930 onward for women. ^2^Full birth cohorts were not included. See text for description.

The Tromsø Study is well suited for sleep studies in the general population, with its unique availability of multiple objective biological measures, as well as current medication use, physical and psychosocial functioning – which will allow research on independent correlates of sleep disturbances.

This protocol describes two main aims:

1) To present descriptive sleep data of a community-based sample representative of the general population of northern Norway.

2) To examine sleep outcome variables against potential explanatory variables examine, both within a cross-sectional approach, as well as retrospectively in a longitudinal study exploring development of sleep characteristic in subjects having attended two or more of the Tromsø studies between 1974 and 2009.

We plan to merge data on an individual level from the Tromsø VI Study with data from the Norwegian Prescription Database (NorPD), which is a national registry including data for all prescription drugs issued at Norwegian pharmacies since 2004 [55]. This will enable research on drug-related sleep complaints.

In addition to the two main aims outlined in this protocol, we are planning a follow-up controlled cohort-study, which will include subjects with sleep complaints recruited in Tromsø VI. This prospective study will aim to investigate possible causal factors of insomnia, and will be described in detail in a later protocol.

## Methods

### Setting

The current study will be performed in a non-institutionalized general population in northern Norway, aged 20 to 90 years. Samples will be drawn in a two-stage procedure, described below.

In the first stage, data from all eligible participants in the Tromsø VI Study will be assessed, in order to describe sleep disturbances and their associated risk factors. In the initial survey, self-reported sleep disturbances will be addressed in questionnaire 1 (Q1), with one single question; "During the last 12 months, how often have you experienced sleep disturbances?", answered with a four-item response: *"never", "1–3 times per month", "once a week" *or *"more than once a week"*. In this initial screening, the selection criterion for sleep disturbances will be *"more than once a week"*.

In the second stage, all participants who report sleep disturbances ("*more than once a week"*) will be invited to a second cross-sectional survey, where sleep complaints and daytime sleepiness will be mapped, using a sleep assessment questionnaire (questionnaire 2 = Q2) described below. A pair-matched control group (matched for age and gender) will be recruited among the remaining participants (those without sleep complaints).

Assuming that the prevalence of sleep complaints in the Tromsø VI Study corresponds to previous estimates for the general population, we expect to include 10 to 15% of the total study population into the sleep complaint group.

In order to analyze possible seasonal effects due to the natural annual light-dark cycle in the artic, we will examine a random half of the participants in the sleep complaint group and control group with Q2 in the part of the year with the highest levels of environmental light (May-June), and examine the remaining participants with Q2 in the part of the year with the lowest levels of environmental light (November-December).

### Questionnaires

All participants in Tromsø VI will be asked to complete an extensive questionnaire (Q1), including demographic data, socioeconomic variables, subjective health perception, quality of life, current medical diagnoses and symptoms, use of prescription drugs and OTC-drugs, use of and satisfaction with health services. In addition, biological markers (i.e. blood samples, height, weight, blood pressure) will be collected.

The sleep assessment questionnaire (Q2) will among other items include the Epworth Sleepiness Scale (ESS) and the Pittsburgh Sleep Quality Index (PSQI).

The ESS [8] is useful in measuring the frequency of daytime sleepiness, and has been used in numerous studies [56-60]. ESS evaluations have demonstrated significant association between daytime sleepiness and insomnia [59], and have allowed quantitative analysis of the effects of pharmacologic and non-pharmacologic therapies on daytime sleepiness in poor sleepers [56,60]. Subjects are instructed to rate the chance of dozing off or falling asleep in eight different "real-life" circumstances. Grading of each item ranges from 0 to 3 and the maximum total score reaches 24. Values over 10 are regarded as indicative of excessive daytime sleepiness [8,61]

The PSQI is a self-rated questionnaire assessing sleep quality and disturbances lasting for more than 1 month [62]. This subjective measure has been validated against polysomnography (PSG) [62-64], which is considered the gold-standard for measuring sleep disturbances [65]. The PSQI is now one of the most commonly used assessments for the evaluation of insomnia severity in sleep research [65,66], and its validity has been demonstrated for patients with mental disorders [62,67], for patients with different somatic diseases [68], and for healthy elderly subjects [63].

Sleep assessment tools, such as ESS and PSQI, are usually treated as binary variables in large population-based studies, according to their cut-off values for "normal" vs. "insomnia". For clinicians, however, a more accurate grading of insomnia is usually needed and the scales are used as continuous variables in order to grade insomnia into e.g. "mild", "moderate" or "severe".

In addition to the PSQI and ESS, Q2 will also address presence of restless legs, nightmares, shift-work and self-reported strategies for coping with insomnia.

### Population

The Norwegian city of Tromsø is situated 70° north of the equator and north of the Arctic Circle. It is the largest city in northern Norway, with approximately 60.000 inhabitants. The aims of the repeated Tromsø studies (Table 2) are to investigate determinants for chronic diseases and to identify potentially modifiable determinants that may contribute to the development of preventive strategies.

In 2001, the size of the study population was 8.130 persons, which was considerably less than most previous Tromsø studies. This was a result of a steered invitation policy to those individuals attending two sub-populations in the 1994/1995 Study.

The size of the population to be invited in the forthcoming Tromsø VI Study is still not established, but is expected to be about 12.000 in the age from 20 to 90 years.

### Outcome measures

Outcome measures in the first part of this study will be the prevalence of sleep disturbances as reported in the initial questionnaire (Q1) containing a four-item response option, where sleep disturbance is defined as the average occurrence of sleep problems *"more than once a week"*.

In the second part of the study, sleep characteristics will be further mapped by a sleep questionnaire (Q2), sent by mail to subjects in the sleep complaint group and in an approximately equal-sized control group. Recruitment, pair matching, questionnaire logistics (including reminders) will be handled by the Tromsø Study's central body, situated at the University of Tromsø. Outcome measures are summarized in Table 3.

**Table 3:** Sleep outcome variables, potential explanatory variables and their pre-specified associations in the Tromsø Study

*Sleep outcome variables against potential explanatory variables*	*Pre-specified associations*	*References*
Initial screening of all eligible participants in the Tromsø Study:		
• Prevalence of sleep disturbances reported in the initial questionnaire (Questionnaire 1) containing a four-item response option, where sleep disturbance is defined as the average occurrence of sleep problems *"more than once a week"*.	10 to 50% in the adult population.	[2, 76]
• Sleep disturbances and their possible relationships, including demographic variables, health-related variables, and lifestyle and socioeconomic variables, and biological markers.	Increased risk for impairments in health, decreased quality of life and increased healthcare utilization.	[19, 65, 77-80]

Further mapping with validated sleep assessment questionnaires among subjects with sleep complaints selected in the initial screening and their control group:		
• Sleep outcome variables related to the use (dose and frequency) of specific prescription drugs, derived from the Norwegian Prescription Database (NorPD).	Frequent use of hypnotics and over-the-counter (OTC) medications.	[36, 37]
• Sleep outcome variables related to socioeconomic variables.	Insomnia related to unemployment and socioeconomic deprivation, more common in women, elderly and individuals living alone.	[81, 82]
• Sleep outcome variables and excessive daytime sleepiness related to objective medical diagnosis, such as diabetes and coronary disease.	A possible link between short sleep, diabetes mellitus, coronary heart disease and all over mortality.	[21, 25-28]
• Sleep outcome variables related to the diagnosis of restless legs.	Relation between symptoms of restless legs and insomnia	[15]
• Sleep outcome variables related to self-reported complaints of musculoskeletal symptoms.	Relation between symptoms of musculoskeletal symptoms and insomnia	[83]
• Sleep outcome variables related to self-reported psychiatric symptoms	Elevated risk of depression and anxiety disorders in people with persistent insomnia	[58, 77, 84-86]
• Sleep outcome variables related to self-reported frequency of nightmares.	Possible association between nightmares and being a woman, feeling depressed after a poor night's sleep, and complaining of a long sleep latency.	[87]
• Sleep outcome variables related to excessive daytime sleepiness and reported actual sleep length.	Daytime sleepiness inversely related to hours of sleep and positively related to the ease of falling asleep at night, especially among young adults.	[88, 89]
• Sleep outcome variables and excessive daytime sleepiness related to subjective perceptions of pain	Chronic pain populations are more likely to experience chronic insomnia, sleep maintenance problems, and/or nonrestorative sleep.	[17, 35, 90]
• Sleep outcome variables and excessive daytime sleepiness related to doctor-seeking behaviour.	Although insomnia is related to more consultations with GPs, many individuals hesitate to consult their doctor about insomnia	[72, 82]

### Statistics

Below, we describe the most probable statistical approaches in the evaluation of associations between self-reported sleep disturbances and exposures. However, in the course of analyses, and with further knowledge of the actual data set, the statistical approach may be supplemented and/or modified. SPSS version 14.0 will be used for statistical analyses. STATA 10 is used for sample size and power calculations.

Age adjustments in groups of subjects with sleep complaints will be performed by the direct method with the Norwegian population serving as standard population.

We plan to carry out series of tabular analyses, tabulating our outcome variables against potential explanatory and confounding variables. These tabulations will guide further analyses. A matched regression analysis will be done using conditional logistic regression in SPSS. For each model we will compare the results of the univariate analyses to those of multivariate analyses. We aim to construct credible parsimonious explanatory models, which will be critiqued using residual analysis, and the Hosmer-Lemeshow goodness of fit test.

Relevant variables that reach significance in bivariate analyzes, will be entered into a multiple logistic regression analyses to determine their influence on sleep outcome variables.

Some of the variables might have missing values. One possible way to deal with missing values is by multiple imputation. To include most of the observations in the model, the missing values may be included after an imputation procedure in SPSS, where the missing value is replaced by the mean of the non-missing values for that variable.

From the first initial screening to the subsequent mapping with the extensive sleep-questionnaire, it may happen that some subjects defined as "cases" reach normal values and vise versa. These subjects and their matched control/case will be excluded from later analyses.

We have calculated sample sizes for three main variables in this study: PSQI, EES and BMI (*p *value for significance = 0.05 (two-sided) and power = 90 %). Ideally, the size of the effect to be detected should be base on clinical judgments, but this is not always straightforward. The effect should be large enough to be clinically important but not so large that it is implausible. We have based our estimates of clinical effects on previous studies of insomniacs compared to healthy controls for PSQI [69], ESS[70]and BMI [20]. We have calculated the standard deviations for each variable (PSQI = 4.58, ESS = 5.9 and BMI = 5.7), and decided that clinically relevant differences for these variables are PSQI > 2, ESS > 2 and BMI > 1. The necessary sample sizes (number of pairs) to detect significant differences between cases and controls are; PSQI = 56 pairs, ESS = 92 pairs and BMI = 342 pairs. Thus, for the three variables above, the planned study seems to be large enough to detect differences between groups.

### Recruitment and randomization

Recruitment of participants to the Tromsø VI Study will be carried out of the study's central body, situated at the Medical Faculty, University of Tromsø. The present plan is to invite the following subgroups in the county of Tromsø:

• A 10% random sample of all inhabitants aged 20 to 39 years.

• All inhabitants aged 40 to 42 years.

• A 10% random sample of all inhabitants aged 43 to 59 years.

• All inhabitants aged 60 to 79 years.

• All inhabitants older than 80 years who have attended at least one of the previous Tromsø studies.

This will comprise a total population of approximately 15.000 persons. An attendance of 80% would imply a study population of approximately 12.000 persons.

### Ethics and data security

Before the recruitment in the current study, all participants will be informed about the objectives for the study, including that their participation is voluntarily and that they are granted full anonymity. A written informed consent will be obtained from all participants. The study will be presented for The Regional Committee for Research Ethics and for The Norwegian Social Science Data Services (NSD).

## Discussion

Sleep disturbances cause great impairment in quality of life [71-73]. The degree of impairment correlates with severity of sleep disturbance and may even rival or exceed the impairment in other chronic medical disorders, like DM, arthritis and heart disease [74]. A wide range of health aspects may be compromised, such as occupational adjustment, physical and social functioning and mental health. Individuals with serious sleep disturbances report diminished energy, concentration, and memory disturbance [18,74,75].

Despite ongoing research, many challenges remain in the characterization of sleep disturbances and its correlates [65]. Retrospective epidemiologic studies have not fully defined sleep disturbances' natural history and pathology [65]. Population-based surveys often yield observational and/or subjective patient-reported data, and there is a remaining need to include sufficient analysis of objective assessments, confounding and/or risk factors and the presence/absence of co-morbidities [65]. Future mapping of the biological dimensions, natural history, as well as the behavioral and drug-related aspects of sleep disturbances in representative population samples is clearly needed.

## Competing interests

The authors declare that they have no competing interests.

## Authors' contributions

AF had the original idea for the study, and prepared the initial draft of the study protocol. All authors have participated in the planning of the study and in the preparation of the final manuscript.

## Pre-publication history

The pre-publication history for this paper can be accessed here:


